# Relationship between Oxytocin and Osteoarthritis: Hope or Despair?

**DOI:** 10.3390/ijms222111784

**Published:** 2021-10-29

**Authors:** Stephanie Ferrero, Ez-Zoubir Amri, Christian Hubert Roux

**Affiliations:** 1 Rheumatology Department, Hospital Pasteur 2 CHU, 06000 Nice, France; ferrero.s@chu-nice.fr; 2 Inserm, CNRS, iBV, Université Côte d’Azur, 06000 Nice, France; Ez-Zoubir.Amri@unice.fr

**Keywords:** oxytocin, osteoarthritis, adipocytes, chondrocytes

## Abstract

Oxytocin (OT) is involved in breastfeeding and childbirth and appears to play a role in regulating the bone matrix. OT is synthesized in the supraoptic and paraventricular nuclei of the hypothalamus and is released in response to numerous stimuli. It also appears to be produced by osteoblasts in the bone marrow, acting as a paracrine–autocrine regulator of bone formation. Osteoarthritis (OA) is a disease of the whole joint. Different tissues involved in OA express OT receptors (OTRs), such as chondrocytes and osteoblasts. This hormone, which levels are reduced in patients with OA, appears to have a stimulatory effect on chondrogenesis. OT involvement in bone biology could occur at both the osteoblast and chondrocyte levels. The relationships between metabolic syndrome, body weight, and OA are well documented, and the possible effects of OT on different parameters of metabolic syndrome, such as diabetes and body weight, are important. In addition, the effects of OT on adipokines and inflammation are also discussed, especially since recent data have shown that low-grade inflammation is also associated with OA. Furthermore, OT also appears to mediate endogenous analgesia in animal and human studies. These observations provide support for the possible interest of OT in OA and its potential therapeutic treatment.

## 1. Introduction

Osteoarthritis (OA) is the most common form of arthritis and a leading cause of impaired mobility in the elderly population [1]. It is a major public health problem in an ageing and increasingly obese worldwide population, and the incidence of OA could increase in the coming years. Therapeutics for OA remain limited, with a constant increase in the cost of care, especially for lower limb localization [2], despite notable advances in understanding its physiopathology. However, recent advances in the epidemiologic and basic research on OA have permitted the differentiation of clinical phenotypes based on the following risk factors: aging, trauma, heredity, obesity, and metabolic syndrome [2,3]. The classification of OA into different phenotypes provides another lens to this complex disease and explains its different evolutionary trajectories depending on various etiological factors. This also suggests the use of specific therapies depending on the phenotype. These observations suggest that there is no longer one but several types of OA.

Alterations in the expression of different signaling pathway-related molecules, such as the TGF-β superfamily of proteins, Wnt/β-catenin, Notch, and Indian Hedgehog (Ihh) have been shown to contribute to the development and progression of OA by primarily inducing catabolic responses [3,4,5,6,7,8,9,10,11]. Such responses include the upregulation of inflammatory mediators that lead to cartilage extracellular matrix (ECM) degradation via increased expression of matrix metalloproteinases (MMPs) and a disintegrin and metalloproteinase with thrombospondin motifs (ADAMTs) [12,13,14,15,16]. OA was previously defined as a non-inflammatory arthropathy, but it is now well-recognized that it affects the joint as a whole, confirming an inflammatory component of this disease.

Oxytocin (OT), a neurohypophyseal hormone discovered in 1906, is synthesized in the paraventricular and supraoptic nuclei of the hypothalamus and is secreted by the posterior pituitary into the general circulation [17,18,19,20,21]. It plays an essential role in reproduction, acting on the smooth muscles of the uterus and the muscles of the mammary glands, particularly during and after childbirth. OT exerts its pharmacological properties by activating specific receptors present in multiple tissues of the body [18]. A growing body of evidence has shown that OT contributes to modulating several functions, such as social recognition, trust, anti-nociception, anti-inflammation, stress reduction, sexuality, and generosity [21,22,23,24,25]. It has also been shown to be produced by peripheral tissues, such as adipocytes, osteoblasts, muscles, testes, ovaries, heart, lung, and vascular tissue [17,18,19,26,27].

In addition, OT receptors (OTRs) have been shown to be expressed on human chondrocytes [27,28], osteoblasts [29], adipocytes, vasculo-endothelial cells [20], monocytes and macrophages [30], and brain cells [31]. Together, the inflammatory status and the expression of OTRs in previous studies have suggested that OT may play a role in OA. Furthermore, multiple studies have shown that OT may also have anti-inflammatory and antioxidant properties and regulate immune and anti-inflammatory responses. For example, OT has been shown to inhibit the secretion of pro-inflammatory cytokines, such as TNF-α, interleukin (IL)-6, IL-1b, glutamate, nitric oxide (NO), and reactive oxygen species (ROS) [32,33].

Interestingly, these parameters show that there are different inflammatory factors implicated in the pathophysiology of OA. OA is now known to be a low-grade inflammatory disorder of the joint as a whole, with inflammation driving several pathological changes [34]. As a progressive joint degenerative disorder, OA is characterized by cartilage damage, changes in the subchondral bone, osteophyte formation, and muscle weakness.

Inflammatory cytokines (TNF-α, IL-6, etc.) play an important role in chondrocyte catabolism at the cartilage level. Matrix-degrading enzymes are considered to play key roles in the degradation of cartilage matrix proteins and MMPs [35,36,37]. MMP expression is regulated by transcription factors, such as activator protein-1 (AP-1), PEA3, RUNX2, and nuclear factor (NF)-κB [38]. Mutations in the AP-1 site completely inhibited the induction of MMP expression. AP-1 contains members of the Fos and Jun families of proteins, where c-Fos/AP-1 directly controls MMP expression by binding to the AP-1 site on the promoters of inflammatory cytokines and MMP genes [39,40,41]. OT activates the MAPK cascade via different pathways, including the trans-activation of receptor tyrosine kinases and possibly different G protein-linked pathways, leading to c-Fos and c-Jun induction [42].

Moreover, Wu et al., showed that OTR expression in primary chondrocytes from patients with OA was reduced compared to that in chondrocytes from control patients [28]. That study showed a reduced expression of the OTR in response to TNF-α treatment, the ability of OT to reverse TNF-α-induced Col II degradation in a dose-dependent manner, and that OT attenuated TNF-α-induced MMP-1 and MMP-13 expression, as well as JAK2/STAT1 activation. Importantly, the inhibitory effect of OT on TNF-α-induced Col II degradation was found to be dependent on OTR. These results highlight the potential of OT in maintaining cartilage integrity. In a previous study, we showed that OT stimulates chondrogenesis in vitro [43]. We observed an increase in glycosaminoglycan content in the extracellular environment following OT treatment. OT induced an increase in the expression of aggrecan, cartilage oligomeric matrix protein (COMP), and SRT-related HMG-box gene 9 (Sox9). In contrast, the expression of the fibrous tissue marker Col A1 was downregulated. Moreover, we found that administration of OT attenuated the effects of IL-1β, as shown by the reduction in ADAMTS-4 mRNA transcript levels; thus, OT may modulate chondrogenesis. A recent study also showed that OT controls chondrocyte matrix degradation through the downregulation of metalloproteinases mRNA expression, strongly supporting the role of OT in the physiopathology of OA [28].

## 2. The Subchondral Bone

The subchondral bone is an intricate structure consisting of two distinct anatomic entities: the subchondral bone plate and subchondral trabecular bone, both of which have a close biomechanical and biochemical relationship with the overlying cartilage [44]. The subchondral bone plate has a marked porosity. It is filled by channels that directly link the articular cartilage and subchondral trabecular bone, allowing crossover communications by prostaglandins, leukotrienes, various growth factors, and inflammatory parameters. A surprisingly high number of arterial and veinous vessels, as well as nerves, penetrate through these channels and send tiny branches directly into calcified cartilage. The subchondral trabecular bone exerts important shock-absorbing and supportive functions in normal joints and may also be important for cartilage nutrient supply and metabolism [45].

The subchondral bone plays a vital role in the pathogenesis of OA. Strong evidence associates subchondral bone alterations with cartilage damage and loss in OA. Subchondral sclerosis is widely considered to be a prominent feature of late-stage OA. At the same time, early-stage OA is characterized by a thinning subchondral plate with increased porosity and deteriorated subchondral trabeculae with decreased bone density [45]. Thus, bone-altering diseases such as osteoporosis could have consequences in the cartilage. For many years, a possible link between osteoporosis and osteoarthritis has been discussed. They are indeed two entities related to age and genetic and environmental factors, including estrogen deficiency [46,47]. They both involve similar cell populations: osteoblasts and chondrocytes, derived from a common progenitor, the mesenchymal stem cell [48,49,50].

It is now widely recognized that the pituitary gland-bone axis plays an essential role in the endocrine regulation of the skeleton. In particular, different groups elucidated the role of pituitary hormones, such as the FSH, GH, prolactin, and OT in the regulation of bone homeostasis [51,52,53,54]. In addition, experiments performed in mice haploinsufficient for pituitary hormones showed decreased hormone levels on their receptors, seriously affecting the skeleton. However, the primary target organ could remain intact, indicating that the bone is more sensitive to the control of pituitary hormones [51,55,56,57]. Indeed, in a study in lactating mice, the bone was more sensitive to the action of OT than the breast because haploinsufficient OT^+/−^ or OTR^+/−^ mice showed profound osteopenia [58]. Consequently, mice deficient in OT or OTR showed profoundly impaired bone formation.

Given that calcium is mobilized from the maternal skeleton during late pregnancy and lactation, the hypothesis that the hormone regulating these functions might also control skeletal homeostasis led to the discovery of a direct anabolic action of OT on the skeleton [51]. A hypothesis on the central action of OT has already been discussed, and one study performed intra-cerebro-ventricular OT injections in mice to demonstrate improvement in bone anabolism. The failure of this study to prove the above hypothesis suggests that the action of OT on the skeleton is concentrated on the more peripheral regions [51]. This result could also be explained by the location of the receptors in the peripheral tissues. It has been shown that OT acts on the subchondral bone, an important structure involved in the pathophysiology of osteoarthritis [58]. Changes in the subchondral bone and the actions of osteoblasts during OA are also well-known [59].

Indeed, studies have shown that OT can promote osteoblastogenesis in human adipocyte and bone marrow mesenchymal (hMADS and hBMS) cells [29,60,61]. In addition, osteoblasts and osteoclasts express OT receptors (OTRs), the stimulation of which increases bone mass [29,30,31]. Indeed, in a study on rats published by our team [30], we have shown that intraperitoneal injection of OT could decrease serum calcium levels and NF-κB expression and increase osteoprotegerin levels and an increase in bone remodeling. Another study also showed that subcutaneous injection of OT in ovariectomized mice could play a role in bone microarchitecture, strengthening it through the control of osteoblast-adipocyte balance. In the same study, a significant decrease in OT levels was observed in osteoporotic postmenopausal women than non-osteoporotic postmenopausal women, proving the strong link between OT and bone microarchitecture [29]. These different elements, showing the action of OT on bone, reinforce the idea of the potential role of this hormone in osteoarthritis, given the important synergies between subchondral bone and cartilage.

## 3. The Muscle

Another joint structure that plays a role in OA is the muscle. The progressive loss of periarticular muscle mass and function has consequences on joint stability and health. Muscle wasting is inevitably associated with aging, and notable weakness in the quadriceps muscle was observed early in the disease process of OA and may even precede disease onset [62,63]. Alterations in the cellular and molecular properties of the quadriceps muscle regarding myofiber atrophy, reduction in muscle quality, and defective muscle regeneration in adults with moderate knee OA have been reported [64]. It seems that patients with OA had fewer Type I fibers and more hybrid IIa/x fibers compared to healthy controls.

Myoblasts express OTRs. Elabd et al., showed in their study on mice that OT, a key age-specific systemic regulator, supports the productive repair and maintenance of skeletal muscle and acts directly on muscle stem cells in vitro and in vivo. The pro-myogenic effect of OT was mediated by MAPK/ERK signaling [60], a pathway implicated in the pathophysiology of OA. A deficiency in skeletal muscle regeneration observed in aged mice was associated with increased fibrotic tissue formation [65,66,67]. Elabd et al., also showed that the fibrotic index was higher in older mice than in younger mice. Ectopic OT treatment significantly decreased the fibrotic index in older mice, whereas administration of OT antagonists increased fibrosis in young mice. Moreover, subcutaneous injection of OT improved muscle regeneration in old mice to a level comparable to that in young mice [60]. These data suggest that the age-related decline in OT is associated with a reduced regeneration of muscle fibers, paving the way for an interest in OT treatment to counteract muscle degradation.

Further intracellular signaling studies in human and mouse myoblasts associated the observations in the OT-mediated promotion of myogenic cell proliferation with extracellular signal-regulated kinase 1/2 phosphorylation in the MAPK/extracellular signal-related kinase pathway [68]. The binding of OT and OTR-selective agonists (Thr, Gly) to the OTRs in human myoblast cultures was also shown to promote myoblast fusion [68], substantiating prior observations of OT-mediated myogenic differentiation in murine L6-C5 skeletal muscle cells [69].

## 4. The Metabolic Syndrome and Adipocytes

Recent evidence indicates that OT enhances glucose uptake and lipid utilization in adipose tissue and skeletal muscle, suggesting that a dysfunction in the OT system could underlie insulin resistance and dyslipidemia pathogenesis. The relationship between OA and metabolic syndrome has also been extensively studied. Metabolic syndrome refers to a cluster of metabolic abnormalities, including obesity, insulin resistance, dyslipidemia, hyperglycemia, and hypertension. OA and diabetes frequently co-exist in patients by chance due to their high prevalence and shared risk factors, such as obesity and aging. Prolonged hyperglycemia, both in fasting and post-prandial states, leads to the production of advanced glycated end products (AGEs), oxidative stress, and low-grade inflammation, eventually resulting in damage to the vasculature, mainly in the heart, kidneys, eyes, nerves, and other tissues [70]. Meta-analyses confirmed an association between osteoarthritis and cardiovascular diseases [71]. To explain the link between OA and metabolic syndrome, in vivo models of obesity, diabetes, and dyslipidemia provided evidence of a systemic effect on joints associated with low-grade inflammation and oxidative stress. Obesity-associated inflammation is associated with osteoarthritis severity and may modulate osteoarthritis progression in mouse models. In addition, osteoarthritis synovium from patients with type 2 diabetes showed insulin-resistant features, suggesting that type 2 diabetes may participate in joint catabolism.

Despite evidence of the mechanical impact of being overweight/obese in elucidating lower limb OA in obese subjects with diabetes, their association in hand OA supports the role of diabetes in OA pathogenesis through two major pathways: oxidative stress resulting from chronic hyperglycemia, leading to the overproduction of pro-inflammatory cytokines and AGEs in joint tissue; insulin resistance, which may impact both local and systemic low-grade chronic inflammation. Dysglycemia, including diabetes, is only one factor of metabolic syndrome. Whether other components of metabolic syndrome, including high blood pressure and atherogenic dyslipidemia, together or independently affect OA pathophysiology remains to be explored [72].

Despite conflicting data in humans, metabolic syndrome—the clustering of cardiometabolic risk factors, including central obesity, insulin resistance, dyslipidemia, and hypertension—has been associated with reduced fasting serum OT levels, as reported in large-scale mixed-gender studies, such as that performed by Yuan et al. [73]. In addition, fasting serum OT concentration has been negatively associated with body mass index (BMI), waist circumference, levels of glycosylated hemoglobin (HbA1c), fasting glucose and post-prandial glucose, fasting and post-prandial insulin, TG, the homeostasis model assessment of insulin resistance (HOMA-IR) score, and C-reactive protein, as reported in a study of subjects with normal body weight and those with overweight and/or obesity [73,74].

The relationship between adipose tissue and OA has also been widely studied [75]. Among systemic adipose tissues, subcutaneous adipose tissue is significantly negatively associated with muscle mass and forces and could be related to the presence and progression of OA in the knee. The amount of visceral adipose tissue is associated with an increased cartilage loss and production of pro-inflammatory cytokines, both of which seem to play a role in the pathogenesis of knee OA [75]. Knee local adipose tissue, such as the infrapatellar fat pad, can interact with neighboring tissues and may have a biphasic effect in knee OA [76]. The underlying mechanisms for the role of systemic and local fat in knee OA could be related to biomechanical, metabolic, and inflammatory factors and fat fibrosis, which may have a separate or combined effect in OA.

OT has previously been shown to inhibit adipocyte differentiation and stimulate osteoblast differentiation [77]. Adipose tissue synthesizes and releases adipokines that modulate bone metabolism by directly or indirectly regulating bone formation and resorption. Adipokines, including leptin, visfatin, adiponectin, and resistin, have been demonstrated to have metabolic implications in the pathogenesis and progression of obesity-induced OA by modulating pro- and anti-inflammatory and anabolic/catabolic balance, apoptosis, matrix remodeling, and subchondral bone ossification [78]. Adipokines appear to play an important role in the pathogenesis of metabolic syndromes. Leptin is an adipokine that exerts its action by activating OB-Rb long-form isoform receptors. It primarily regulates food intake, body weight, and energy homeostasis through neuroendocrine functions and influences insulin sensitivity and lipid metabolism. Subjects with metabolic syndrome had higher leptin levels than those without metabolic syndrome [79].

Dong et al., investigated the differential expression of adipokines in patients with knee OA. They found that leptin expression was higher in patients with knee OA and metabolic syndrome than in those with knee OA without metabolic syndrome [80]. These data correlated with the results of the study of Pelletier et al., who demonstrated that serum levels of leptin predict higher cartilage volume loss in the lateral and medial compartments over time [81]. However, several studies have highlighted the difficulty in interpreting this relationship, given the close links between leptin and certain confounding factors such as BMI, age, and sex. Thus, some authors have found an association between leptin levels and the severity of knee OA or pain, but this was not found after adjustment for potential confounding factors [82,83].

Adipocytes also express OT receptors and signaling through these receptors induces lipolysis [84]. OT levels have been shown to be significantly negatively correlated with leptin levels, and to overcome leptin resistance as it decreases body weight and fat mass with an improvement in glucose metabolism [85]. OT administration has also been shown to successfully treat obesity in animal models of leptin deficiency or reduced leptin receptor signaling [86,87]. Two weeks of subcutaneous OT treatment on obese mice resulted in a reduction in body fat, which translated into a reduction in body weight. This effect was mainly observed during the first week of treatment. However, the effect was more prolonged in db/db mice, wherein the weight loss associated with the loss of fat mass was observed over 12 weeks, with the absence of loss of appetite, which could have been observed in ob/ob mice. A better glucose tolerance and insulin sensitivity was found in db/db mice compared to ob/ob mice [88,89]. However, this phenomenon has conflicting results, especially in some ex vivo or in vivo studies.

## 5. OT as a Potential Therapeutic Treatment

In view of these differences, the therapeutic role of OT in human clinical trials is all the more relevant. In a previous study, we showed that the rate of OT was significantly lower in patients with OA than in patients without OA [43].

In this review, we show the potential relationship between OT and osteoarthritis. The presence of OTR in tissues plays an important role in the physiopathology of OA. The potential beneficial action of OT on the cartilage, subchondral bone, muscle, and inflammation, and its link with other risk factors, including metabolic syndrome, are different points of discussion when looking at the applications of OT treatment in OA. Obesity is a major risk factor for OA, and one of our recommendations for the management of OA is weight loss. The anorexigenic effect of OT has been extensively documented in animals [88,90,91,92,93,94]. Systemic administration of OT has been shown to affect appetite, weight gain, glucose homeostasis, and lipid metabolism in animal models [84,85,86,87,88].

In a previous study, we showed that OT administration in rats limited weight gain compared to the controls [43]. However, definitive studies on the anorexigenic effects of OT in humans still lack due to contradictory results. In a pilot study, Zhang et al. [94] showed that the four times daily administration of intranasal OT (24 IU) in nine Asian men and women with obesity (BMI ≥ 28 kg/m^2^) for eight weeks led to a mean BMI reduction of 3.2 ± 1.9 kg/m^2^. The magnitude of weight loss was also greater in subjects with a higher degree of obesity. This pilot study raised more questions than answers.

Most of the available data on the use of OT for OA came from animal studies. In humans, the data available still cannot convincingly suggest the use of OT for OA. However, while OT injections produced an increase in non-esterified fatty acid (NEFA) levels a few days after delivery [95], it seems that administration of 5 IU of OT via quick infusion resulted in decreased NEFA levels in non-pregnant healthy subjects [96]. A more recent study using chronic treatment of obese humans with nasal OT delivery did not significantly modify triglyceride (TG) levels, although there was a trend toward a decrease in this parameter [92]. This pilot study [94] should be analyzed with caution because of the small number of subjects (eleven placebo-treated vs. nine treated with OT), despite randomization. As this is a study presenting results in both animals and humans, the results in mice showed an interesting effect on carbohydrate metabolism and a reversal of insulin resistance. This is very interesting because obesity is often associated with type 2 diabetes. Authors found that while OT treatment did not affect fasting blood glucose or insulin levels, it tended to reduce post-prandial glucose and insulin levels towards the normal ranges. Modifications independent of weight variations suggested that OT could employ a body-weight-independent mechanism to improve glucose and insulin homeostasis. OT treatment significantly reduced serum LDL and cholesterol levels, while it increased serum HDL levels (ref). The effect of 4-week treatment with OT in humans resulted in a body weight reduction of 4.6 ± 3.2 kg. This therapeutic effect continued to improve when the duration of OT treatment increased to 8 weeks, showing that compared to the baseline levels, the body weight of patients dropped 8.9 ± 5.4 kg (*p* < 0.001) [94].

The advantages of using OT in treating obesity can also be further increased, given that OT has social-neuropsychiatric benefits that can aid in controlling metabolic disease and promoting physical activity. OA is one of the most frequently occurring, painful conditions humans can experience. Pain is a major symptom of OA, involving both peripheral and central neurological mechanisms. OA pain is initiated from free axonal endings in the synovium, periosteum bone, and tendons but not in the cartilage.

Nociceptive messages involve neuromediators and regulating factors, such as neuronal growth factor (NGF) and central modifications of pain pathways [97]. In an animal model, OT release suppresses nociception and induces analgesia by affecting inflammatory pain pathways [98]. OT modulates pain by reaching the spinal cord through fast neuronal projections and slow peripheral pathways. Nersessian et al., showed that OT directly targets the pain receptor TRPV1, a non-selective Ca^2+^-permeable cation channel [99]. The analgesic actions of OT have been documented in patients with migraine administered with intranasal OT [100]. OT-promoted pain regulation involves endogenous analgesia [101].

## 6. Conclusions

All these data led us to postulate the role of OT treatment in osteoarthritis despite conflicting results in the management of obesity. Many reasons may explain the discrepant results in the use of OT in obesity treatment, such as the use of different models, doses, and duration of OT treatments. These observations should help us to think about how OT can be used in OA pathology. However, because of the complexity of the physiopathology of this disease, taking into account the action of OT on multiple tissues that play other important roles in osteoarthritis (cartilage, subchondral bone, muscle, adipocytes, etc.) and its anti-inflammatory action, OT may have different effects depending on the osteoarthritis phenotype. It is certain that the metabolic phenotype seems the most attractive. An evaluation of the effect of OT on osteoarthritis pain will probably be one of the more interesting points that need to be considered, as its peripheral and central origin are favorable aspects for its effectiveness. Only clinical studies on humans will allow us to answer these questions. However, unresolved questions will have to be answered, such as the mode of administration of OT, the rate of administration, as well as the dosage to be administered. There are many hopes, with the development of new technologies, that OT can be delivered directly to the target tissues to avoid side effects as well as to circumvent the very short half lifetime of OT.

## Data Availability

This study did not report data.
